# Chemical composition, in vitro antitumor and pro-oxidant activities of *Glandora rosmarinifolia* (Boraginaceae) essential oil

**DOI:** 10.1371/journal.pone.0196947

**Published:** 2018-05-03

**Authors:** Paola Poma, Manuela Labbozzetta, Monica Notarbartolo, Maurizio Bruno, Antonella Maggio, Sergio Rosselli, Maurizio Sajeva, Pietro Zito

**Affiliations:** 1 Department of Health Sciences and Mother and Child Care ‘G. D'Alessandro’(PROSAMI), Pharmacology Unit, University of Palermo, Palermo, Italy; 2 Department of Biological, Chemical and Pharmaceutical Science and Technology (STEBICEF), University of Palermo, Palermo, Italy; University of PECS Medical School, HUNGARY

## Abstract

The biological properties of essential oils have been demonstrated in the treatment of several diseases and to enhance the bioavailability of other drugs. In natural habitats the essential oils compounds may play important roles in the protection of the plants as antibacterials, antivirals, antifungals, insecticides and also against herbivores by reducing their appetite for such plants or by repelling undesirable others. We analyzed by gas-chromatography mass spectrometry the chemical composition of the essential oil of aerial parts of *Glandora rosmarinifolia* (Ten.) D.C. Thomas obtained by hydrodistillation and verified some biological activities on a panel of hepatocellular carcinoma cell lines (HA22T/VGH, HepG2, Hep3B) and triple negative breast cancer cell lines (SUM 149, MDA-MB-231). In the essential oil we detected 35 compounds. The results of the biological assays indicate that essential oil of *G*. *rosmarinifolia* induces cell growth inhibition at concentration-dependent way in all cell line models. This oil does not seem to possess antioxidant activity, while the cytotoxicity of *G*. *rosmarinifolia* essential oil appeared to involve, at least in part, a pro-oxidant mechanism. Our results show for the first time the antitumoral and pro-oxidant activities of *G*. *rosmarinifolia* essential oil and suggest that it may represent a resource of pharmacologically active compounds.

## Introduction

The family Boraginaceae includes more than 2700 species commonly found in cosmopolitan habitats especially in Tropic, Turian (Iran) and Mediterranean regions [1]. Several species from this family has been traditionally used as diuretic, anti-inflammatory, diaphoretic and sedative as well as rheumatic pain and burns [2, 3]. From a chemical point of view, the species are characterised by naphthoquinones and its derivatives, pyrrolizidine alkaloids, phenolic compounds and its derivatives [4].

*Glandora rosmarinifolia* (Ten.) D.C. Thomas (Boraginaceae) [synonyms: *Lithodora rosmarinifolia* (Ten.) I.M. Johnst.—*Lithospermum rosmarinifolium* Ten.] is a perennial shrub growing on rocky limestone habitats and cliffs in Algeria, Sicily and in the Sorrento peninsula (Italy) [5, 6, 7]. *Glandora rosmarinifolia* has been studied from a molecular [5], and morphological point of view [8, 9].

Few papers have been published on the chemical composition of *Glandora* species (e.g. *G*. *diffusa*) [4, 10] but there are no studies on *G*. *rosmarinifolia*.

The aim of this study was to identify the chemical composition of the essential oil of aerial parts (branches with leaves) of *G*. *rosmarinifolia* obtained by hydrodistillation (HD), analysed by gas chromatography-mass spectrometry (GC-MS), and to verify some biological activities on a panel of hepatocellular carcinoma cell lines (HA22T/VGH, HepG2, Hep3B) and triple negative breast cancer cell lines (SUM 149, MDA-MB-231). Both cancer models are highly aggressive with poor prognosis and are characterized by multi-drug resistance to conventional chemotherapeutic drugs [11, 12]. It is therefore desirable to investigate new therapeutic tools to overcome the adverse biological characteristic and drug resistance of these tumor models.

## Materials and methods

### Plant material

Branches with leaves of *G*. *rosmarinifolia* were collected in February 2017 from plants located in the following site: “Raffo Rosso, Monte Cuccio, and Vallone Sagana” (38°11’07”N 13°16’05” E—Sicily—Palermo). The matrices were placed in paper bags and kept at 4 ± 1 °C for 24 hours before the hydrodistillation. A voucher specimen (N° MS 120/2017) was deposited at the Herbarium of the University of Palermo. No specific permits were required for the described location and for the collection of plant material. The plant species used in the present study is not endangered or protected.

### Essential oil isolation

Branches with leaves (64 g) were hand-cut into small pieces (~ 2 cm) and then hydrodistillated for 3 hours in a Clevenger-type apparatus, using *n*-pentane as collection solvent. The oil was dried by anhydrous sodium sulphate, filtered and stored at 4 °C until its chemical analysis and pharmacological tests. The essential oil yield was 49.8 mg (0.08%).

### Gas chromatography-mass spectrometry

The GC analysis was performed in an Agilent 7000C GC system, fitted with a fused silica Agilent HP-5MS capillary column (30 m x 0.25 mm i.d.; 0.25 μm film thickness), coupled to an Agilent triple quadrupole Mass Selective Detector MSD 5973; ionization voltage 70 eV; electron multiplier energy 2000 V; transfer line temperature, 295 °C. Helium was the carrier gas (1 mL min^-1^). The other GC analysis was performed in a Shimadzu QP 2010 plus, single quadrupole GC/MS system, fitted with a Supelcowax 10 capillary column (30 m x 0.25 mm i.d.; 0.25 μm film thickness); ionization voltage 70 eV; transfer line temperature, 280 °C. Helium was the carrier gas (1 mL min^-1^). For both columns, the temperature was initially kept at 40 °C for 5 min, then gradually increased to 250°C at 2°C min^-1^ rate, held for 15 min and finally raised to 270 °C at 10 °C min^-1^. One μL of diluted samples (1/100 v/v, in *n*-hexane) was injected at 250 °C automatically and in the splitless mode; transfer line temperature, 295 °C.

### Identification of compounds

Identification of compounds was carried out using NIST 11, Wiley 9, FFNSC 2, and Adams [13] databases. These identifications were confirmed by Linear Retention Indices (LRI) with those available in literature by SciFinder database. Some of the compounds were also confirmed by comparison of mass spectra and retention times with standard compounds available in laboratory. The retention indices were determined in relation to a homologous series of *n*-alkanes (C_8_-C_30_) injected under the same operating conditions. Component relative concentrations were calculated based on GC peak areas without using correction factors.

### Cell growth assays

Hepatocellular carcinoma cell lines (HA22T/VGH, HepG2, Hep3B) and triple negative breast cancer cell lines (SUM 149, MDA-MB-231) were used. The human hepatocellular carcinoma cell line HA22T/VGH was kindly provided by Professor Massimo Levrero (Laboratory of Gene Expression, Fondazione Andrea Cesalpino, University of Rome “La Sapienza”, Rome, Italy). HepG2 (ATCC^®^: HB-8065^™^) and Hep3B (ATCC^®^: HB-8064^™^) cells were obtained from the American Type Culture Collection (ATCC, Rockville, MD, USA). All the above cell lines were authenticated by short tandem repeat profiling (BMR Genomics, Padua, Italy). The human breast cancer cell lines MDA-MB-231 (ATCC^®^: HTB-26^™^—Rockville, MD, USA) and SUM149 (SUM149PT—Asterand Bioscience Detroit, MI) were kindly provided by Dr. Elda Tagliabue (Molecular Targeting Unit, Department of Experimental Oncology and Molecular Medicine, Fondazione Institute of Hospitalization and Scientific Care, National Cancer Institute, Milan, Italy) and were authenticated using the short tandem repeat profiling method in their Institute facility.

The cells were seeded at 2 × 10^4^ cells/well onto 96-well plates and incubated overnight at 37 °C. After obtaining the cells, the first passage carried out was assigned passage number 1. Cells with a narrow range of passage number (4–6) were routinely tested for Mycoplasma contamination were used for all experiments.

At time 0, the medium was replaced with fresh complete medium supplemented of essential at the indicated concentrations. Following 72 h of treatment, 16 μL of a commercial solution obtained from Promega Corporation (Madison, WI, USA) containing 3-(4,5-dimethylthiazol-2-yl)-5-(3-carboxy methoxyphenyl)-2-(4-sulphophenyl)-2H-tetrazolium (MTS) and phenazine ethosulfate were added. The plates were incubated in a humidified atmosphere at 37 °C in 5% CO_2_ for 2 h, and the bioreduction of MTS dye was evaluated by measuring the absorbance of each well at 490 nm using a microplate absorbance reader (iMark Microplate Reader; Bio-Rad Laboratories, Inc., Hercules, CA, USA). Cell growth inhibition was expressed as a percentage (mean ± SD) of the absorbance of the control cells.

### Anti- and pro-oxidant activity

To evaluate antioxidant activity, we have used DPPH Assay. The antiradical efficiency of the sample was evaluated by the DPPH stable radical method [14, 15]. 100 μL of sample, the essential oil of *G*. *rosmarinifolia* at different concentrations, was added to aliquots (3.9 mL) of a solution made up with DPPH (4.8 mg) in MeOH (200 mL), and the mixture was incubated for 1 h at room temperature in the dark. Then the absorbance was measured at 517 nm using a UV-VIS spectrophotometer. The initial concentration of DPPH was approximately 60 μM. Lower absorbance values of reaction mixture indicate higher free radical scavenging activity. The results were plotted as the percentage of absorbance disappearance at 517 nm [(1—A/A0) × 100] against the amount of sample divided by the initial concentration of DPPH. Each point was acquired in triplicate. ED_50_ corresponds to micrograms of fraction able to consume half the amount of free radical divided by micromoles of initial DPPH. The results were expressed as antiradical capacity (ARC), which is the inverse of ED_50_.

Trolox (6-hydroxy-2,5,7,8-tetramethyl-chroman-2-carboxylic acid) curve was used as the positive control.

Pro-oxidant activity was examined by cell counting, adding N-acetyl-L-cysteine (NAC), an antioxidant molecule, 1 h before essential oil. Data were expressed as mean ± standard deviation (SD) of at least three different experiments performed in duplicate. All the chemicals were supplied by Sigma Aldrich srl, Milan, Italy.

### Statistical analysis

Results of biological assays are given as means ± standard deviation. Statistical analysis was carried out by analysis of variance (one-way ANOVA) followed by Tukey’s test. Statistica ver. 10 (StatSoft Inc. 2011) was used as software for the analyses.

## Results

### Chemical composition

In the essential oil of *G*. *rosmarinifolia*, we detected 35 compounds: 10 aliphatic alkanes (33.5%), 5 monoterpene hydrocarbons (3.6%), 4 diterpene hydrocarbons (25.9%), 3 aliphatic alcohols (2.3%), 3 aliphatic aldehydes (4.5%), 2 aromatic compounds (5.8%), 2 sesquiterpene hydrocarbons (1.5%), 1 aliphatic ester (0.5%), 1 aromatic ether (0.8%), 1 diterpene alcohol (4.1%), 1 irregular terpene ketone (0.5%), 1 monoterpene alcohol (0.5%) and 1 phenolic compound (1.7%) (Table 1). The most abundant compounds (≥ 4.0%) were *m*-camphorene (13.3%), heptacosane (10.7%), nonacosane (6.6%), hydroxy-methyl-naphthoquinone (5.3%; isomer not identified), 2,6-dimethyl-10-(*p*-tolyl)-2,6-(*E*)-undecadiene (4.9%), cembrene C (4.8%) and phytol (4.1%) contributing together 49.7% of the total composition accounting to 85.2%. Eleven compounds were found in amounts between 1.3 and 3.6% and another 17 compounds were detected in amounts < 1% (Table 1).

**Table 1:** Essential oil composition of *G*. *rosmarinifolia*. Compounds belonging to the same chemical class are arranged according to Linear Retention Indices (LRI) of the HP-5MS column.

LRI^a^	LRI^b^	Compound	Relative amount (%)
		***Aliphatic Alcohols***	
862	1381	(*Z*)-3-Hexenol	0.9
1778	2376	Pentadecanol	0.5
1968	2447	Heptadecanol	0.9
		***Aliphatic Aldehydes***	
1001	1285	Octanal	0.3
1104	1388	Nonanal	1.6
1205	1493	Decanal	2.6
		***Aliphatic Alkanes***	
2000	2000	Eicosane	1.3
2100	2100	Heneicosane	3.6
2200	2200	Docosane	0.8
2300	2300	Tricosane	3.6
2400	2400	Tetracosane	0.7
2500	2500	Pentacosane	3.3
2600	2600	Hexacosane	1.7
2700	2700	Heptacosane	10.7
2800	2800	Octacosane	1.2
2900	2900	Nonacosane	6.6
		***Aliphatic Esters***	
2009	2376	Hexadecyl acetate	0.5
		***Aromatic Compounds***	
1452	2056	Precocene I	0.5
1593	2311	Hydroxy-methyl-naphthoquinone^#^	5.3
		***Aromatic Ethers***	
1620	2341	Dill apiole	0.8
		***Diterpene Alcohols***	
2120	2609	Phytol	4.1
		***Diterpene Hydrocarbons***	
1951	2278	2,6-Dimethyl-10-(*p*-tolyl)-2,6-(*E*)-undecadiene	4.9
1965	2223	*m*-Camphorene	13.3
1994	2275	*p*-Camphorene	2.9
2028	2153	Cembrene C	4.8
		***Irregular Terpene Ketones***	
1482	1923	*β*-Ionone	0.5
		***Monoterpene Alcohol***	
1213	1781	Myrtenol	0.5
		***Monoterpene Hydrocarbons***	
933	1017	*α*-Pinene	0.9
993	1161	Myrcene	0.5
1024	1260	*o*-Cymene	0.3
1028	1189	Limonene	1.6
1060	1235	*γ*-Terpinene	0.3
		***Phenolic Compounds***	
1309	2191	4-Vinylguaiacol	1.7
		***Sesquiterpene Hydrocarbons***	
1411	1574	*β*-Caryophyllene	0.9
1447	1645	*α*-Humulene	0.6
**Total**			**85.2**

LRI^a^: Linear Retention Index on HP-5MS column;

LRI^b^: Linear Retention Index on Supelcowax 10 column;

^#^ isomer not identified, EIMS: 189 (13), 188 (100), 173 (43), 145 (33), 131 (8), 127 (10), 116 (8), 117 (20), 115 (38), 91 (17).

### In vitro anticancer and pro-oxidant activity

**Fig 1:**
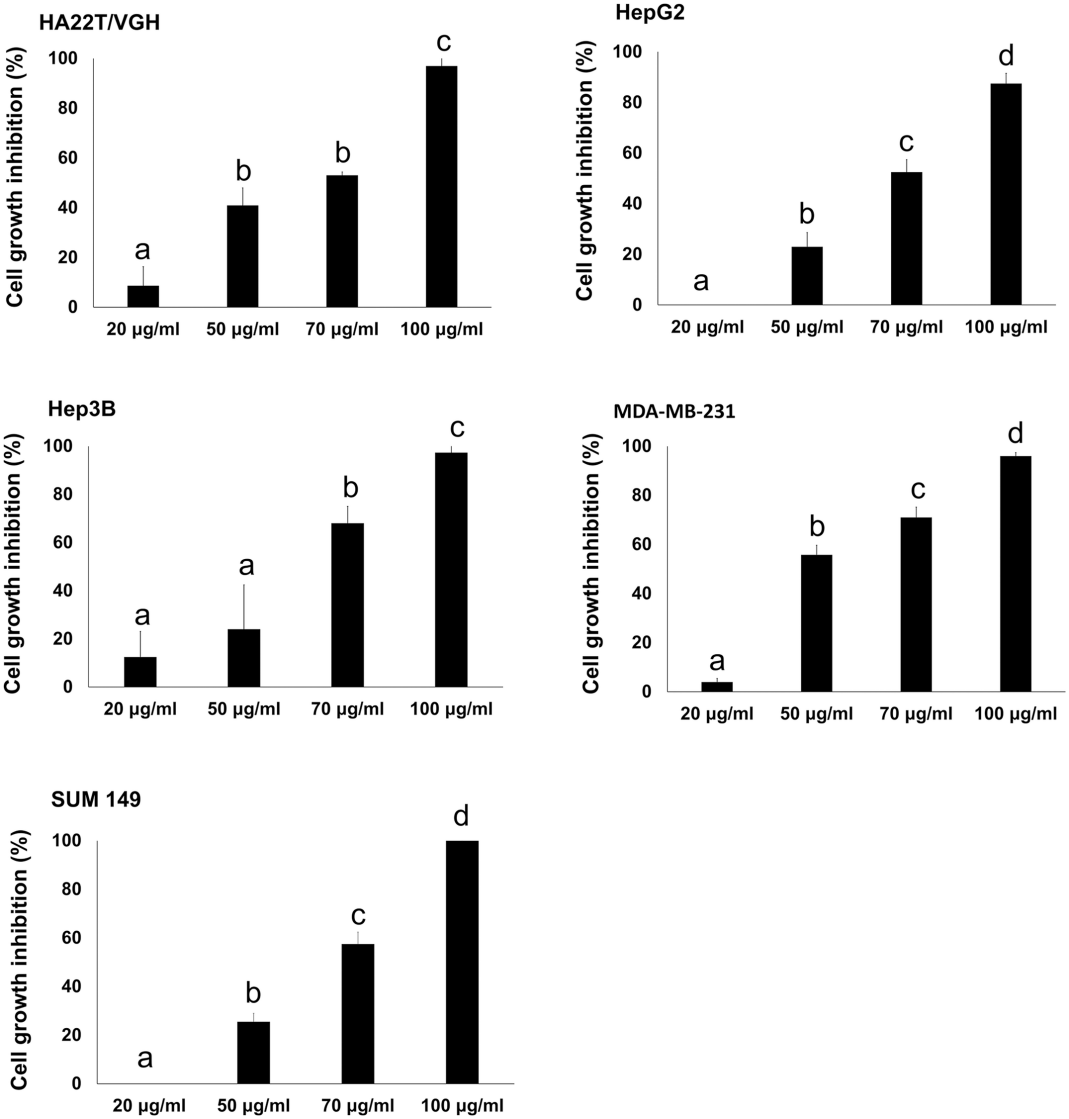
Cytotoxic activity of *G*. *rosmarinifolia* essential oil on cancer cell lines. Cell viability was assessed by MTS. Data are expressed as mean ± standard deviation (SD) of at least three different experiments performed in triplicate. Different letters represent significant differences (*p* < 0.01) in cytotoxic activity among the concentrations of each cell line.

We investigated the cytotoxic activity of *G*. *rosmarinifolia* essential oil by 3-(4,5-dimethylthiazol-2-yl)-5-(3-carboxy methoxyphenyl)-2-(4-sulphophenyl)-2H-tetrazolium (MTS) assay on a panel of hepatocellular carcinoma cell lines (HA22T/VGH, HepG2, Hep3B) and triple negative breast cancer cell lines (SUM 149, MDA-MB-231). As shown in Fig 1, the essential oil induces cell growth inhibition at concentration dependent way in all cell line models. The relative IC_50_ are reported in Table 2.

**Table 2:** IC_50_ values of the five cell lines treated with the essential oil of *G*. *rosmarinifolia*.

Cell line	IC_50_ (mean ± SD)
HA22T/VGH	60.5 ± 2.1 μg/mL
HepG2	65.0 ± 0.0 μg/mL
Hep3B	61.0 ± 5.6 μg/mL
SUM 149	65.0 ± 4.2 μg/mL
MDA-MB-231	46.5 ± 2.1 μg/mL

In order to explain if antitumor activity of this oil could depend on pro-oxidant activity, as in the case of other natural compounds, we performed a separate experiment in which cell lines were treated with the antioxidant N-acetyl-L-cysteine (NAC) at two concentrations, 1 mM and 2 mM before exposure to the essential oil at the corresponding IC_50_ values as reported in Tables 2 and 3. In all tested cell lines a reduction between 27.3% and 38.0% of the cytotoxicity activity of the essential oil was observed, suggesting that the mechanism involves an at least partially pro-oxidant effect (Table 3).

**Table 3:** Results of cell counting analysis in the five cell lines following treatment with antioxidant N-acetyl-L-cysteine (NAC) at two concentrations, 1 mM and 2 mM before exposure to the essential oil at the corresponding IC_50_. Data are expressed as mean ± standard deviation (SD).

Cell lines and treatments	Cell viability, %
***HA22T/VGH***	
+ NAC 1 mM ^a^	96.3 ± 0.3
+ NAC 2 mM ^b,^*	81.3 ± 3.7
+ essential oil of *G*. *rosmarinifolia* 60.5 μg/mL ^c,^*	57.5 ± 2.5
+ NAC 1 mM + essential oil of *G*. *rosmarinifolia* ^ab,^*	84.8± 0.3
+ NAC 2 mM + essential oil of *G*. *rosmarinifolia* ^ab^	90.0 ± 1.0
***HepG2***	
+ NAC 1 mM ^a^	94.0 ± 1.0
+ NAC 2 mM ^a^	90.3 ± 0.8
+ essential oil of *G*. *rosmarinifolia* 65 μg/mL ^b,^*	53.4 ± 4.6
+ NAC 1 mM + essential oil of *G*. *rosmarinifolia* ^a^	89.8 ±1.8
+ NAC 2 mM + essential oil of *G*. *rosmarinifolia* ^a,^*	83.0 ± 4.0
***Hep3B***	
+ NAC 1 mM ^a,^*	86.5 ± 0.5
+ NAC 2 mM ^a,^*	84.0 ± 0.0
+ essential oil of *G*. *rosmarinifolia* 61 μg/mL ^b,^*	54.5 ± 1.5
+ NAC 1 mM + essential oil of *G*. *rosmarinifolia* ^a,^*	84.5 ± 0.5
+ NAC 2 mM + essential oil of *G*. *rosmarinifolia* ^a,^*	81.0 ± 5.0
***MDA-MB-231***	
+ NAC 1 mM ^a,^*	89.5 ± 0.5
+ NAC 2 mM ^a,^*	86.0 ± 3.0
+ essential oil of *G*. *rosmarinifolia* 46.5 μg/mL ^b,^*	49.5 ± 0.5
+ NAC 1 mM + essential oil of *G*. *rosmarinifolia* ^a,^*	87.5 ± 1.5
+ NAC 2 mM + essential oil of *G*. *rosmarinifolia* ^c,^*	77.0 ± 0.0
***SUM 149***	
+ NAC 1 mM ^a^	95.5 ± 0.5
+ NAC 2 mM ^a^	94.0 ± 1.0
+ essential oil of *G*. *rosmarinifolia* 65 μg/mL ^b,^*	55.8 ± 2.8
+ NAC 1 mM + essential oil of *G*. *rosmarinifolia* ^a,^*	91.5 ± 0.5
+ NAC 2 mM + essential oil of *G*. *rosmarinifolia* ^a,^*	89.0 ± 0.0

Different letters (a, b and c) in the column of the cell lines and treatments represent significant differences among the treatments of each cell line;

* differences when treatments are compared to the control; *p* < 0.05.

DPPH (2,2-diphenyl-1-picrylhydrazyl) reduction assay confirmed that essential oil does not possess antioxidant activity, in fact on the contrary to known antioxidants as Trolox (ED_50_ 117.5 μg/ml, ACR 0.0085), it was not identified the efficient dose (ED_50_) of the essential oil of *G*. *rosmarinifolia* (Table 4).

**Table 4:** Results of antioxidant activity performed with DPPH method (DPPH free radical scavenging activity).

Compound	ED_50_	ACR (1/ED_50_)
Trolox	117.5 μg/ml	0.0085
Essential oil of *G*. *rosmarinifolia*	>500 μg/ml	-

## Discussion

Essential oils have been used for centuries in folk medicine and in the last few years the biological properties of different essential oils have been demonstrated. Their use in the treatment of pain, inflammation, viral diseases and cancer, enhancement of the bioavailability of other drugs, as well as repellent/insecticidal properties against stored product and crop insects were confirmed [16]. According to Bakkali *et al*. [17] in natural habitats the essential oils compounds may play important roles in the protection of the plants as antibacterials, antivirals, antifungals, insecticides and also against herbivores by reducing their appetite for such plants or by repelling undesirable others.

Our chemical composition indicates that the compounds found in *G*. *rosmarinifolia* essential oil potentially have several biological roles. Among others, diterpenes contributing to 30% of *G*. *rosmarinifolia* composition could have beneficial effects as bactericides, as protection against free radical cell damages, as anti-inflammatory and in the treatment of cancer, among others [18]. As reported by Islam [19] diterpenes may act as anticancer alone or in combination with other diterpenes or anticancer drugs with synergic effects. In particular, phytol, occurring in *G*. *rosmarinifolia* essential oil to 4.1%, can induce apoptosis in human gastric adenocarcinoma cells and its cytotoxic activity has been demonstrated *in vitro* for several tumor cell lines [20]. Moreover, the diterpenes have also evident actions against toxic substances that cause cytogenotoxic damages in biological systems [19]. In an ecological context, they may confer to plants resistance against pests or pathogens [21].

A hydroxy-methyl-naphthoquinone is also among of the main compounds (5.3%) found in the essential oil of *G*. *rosmarinifolia*. It was recognized as a naphotoquinones belonging to hydroxy-methyl-naphthoquinone since its mass spectrum and its linear retention indices are very similar to those of other known isomers of hydroxyl-methyl-naphthoquinones (e.g. plumbagin, 7-methyl-juglone). It is interesting to note that naphthoquinones are biosynthesized in several plant families (e.g. Plumbaginaceae, Juglandaceae, Boraginaceae, Iridaceae, Verbenaceae, Scrophulariaceae, Gentianaceae, Droseraceae, and Euphorbiaceae, among others) as well as in algae, fungi, some animals and also as products of the metabolism in some bacteria [22]. From an ecological point of view, naphthoquinones act as chemical defense for many plants and several naphthoquinones are essentials in the biosynthesis of many molluscicidal and insecticidal compounds (e.g. 2-hydroxy-1,4-naphthoquinone derivatives) [22]. Furthermore, naphthoquinones and their derivatives are compounds with important pharmacological activities such as anticancer, antibacterial, antifungal, antiviral and anti-parasitic (e.g. antimalarial and antileishmania) [23, 24]. Pharmacological activities showed by naphthoquinones and derivatives can be explicated through two mechanisms: (1) as pro-oxidants, reducing oxygen to reactive oxygen species (ROS); and (2) as electrophiles, forming covalent bonds with tissue nucleophiles [25, 26]. According to López *et al*. [22], hydroxy-naphthoquinones and their derivatives are promising compounds for treating disease and for pest control. Several natural compounds exert its antitumor activity through anti or pro-oxidant mechanisms. In the present study, *G*. *rosmarinifolia* essential oil induces a strong suppressive growth effects in all tested cancer cell lines, probably through an increase in free radical generation due to diterpenes and hydroxy-methyl-naphthoquinone (Table 1) which are the main compounds found in this oil [19, 25, 26].

Our previous data on the same tumor cellular models, demonstrated that the redox perturbation due to natural compounds led to inhibition of NF-κB, a transcriptional factor often constitutively activated in many type of cancers and an important target for antitumor therapy [11, 27]. It would be interesting to assess the chemical structure of the tentatively identified hydroxy-methyl-naphthoquinone. Further studies should be also necessary to confirm if the diterpenes and this hydroxy-methyl-naphthoquinone are NF-κB inhibitors in these cancer models and to evaluate their possible use as anticancer drugs.

## Conclusions

Our results show for the first time the antitumoral and pro-oxidant activities of *G*. *rosmarinifolia* essential oil. *G*. *rosmarinifolia* thus represents a resource of pharmacologically valuable compounds and we speculate that its anticancer property could be linked to the high content in diterpenes and naphthoquinone. However, further experiments must be conducted to identify molecular pathway in which the oil compounds are involved.
